# Fatty liver disease and pancreatic inflammation—A lethal combination?

**DOI:** 10.1002/ueg2.12400

**Published:** 2023-05-11

**Authors:** Sara Regnér, Kristina Önnerhag, Hanna Sternby

**Affiliations:** ^1^ Surgery Research Unit Department of Clinical Sciences Malmö Lund University Malmö Sweden; ^2^ Department of Surgery and Gastroenterology Skåne University Hospital Malmö Sweden; ^3^ Gastroenterology Research Unit Department of Clinical Sciences Malmö Lund University Malmö Sweden

**Keywords:** acute pancreatitis, metabolic dysfunction‐associated fatty liver disease (MAFLD), prediction of severity, risk factors

Acute pancreatitis (AP) is a common disorder causing considerable morbidity and mortality risks for the subgroups suffering from more severe forms of the disease.^1^ Multiple risk factors contributing to disease severity have been identified, among them the components of the metabolic syndrome.^2^, ^3^, ^4^ Additionally, fatty liver disease, despite etiology, has been shown to worsen the outcome of AP and inclusion of this entity into prognostic scoring systems has been suggested.^5^, ^6^ For patients with moderate or severe AP, fast and efficient treatment of complications is a prerequisite to keep mortality as well as long‐term morbidity levels low. These patients often require treatment at an intermediate or intensive level of care even though they initially have clinical symptoms similar to patients with mild disease. As there is still a lack of sufficiently correct predictive biomarkers, the identification of risk factors is crucial for clinical guidance and optimal management of the AP patients.

The incidence of both AP and non‐alcoholic fatty liver disease (NAFLD) is on the rise.^7^, ^8^, ^9^ NAFLD nearly affects a fourth of the adult population globally and is additionally associated with the metabolic syndrome featuring a bidirectional interaction.^9^, ^10^, ^11^ In 2020, the criteria for NAFLD were redefined and a new entity, metabolic dysfunction‐associated fatty liver disease (MAFLD), was proposed from an international expert consensus.^12^ The objective was to implement more distinct disease definitions, thus facilitating both routine clinical diagnostics but also trial inclusion and comparison of research results. A positive diagnosis of MAFLD requires the detection of hepatic steatosis in combination with overweight or type 2 diabetes mellitus or the presence of at least two metabolic risk abnormalities. It does not require the exclusion of alcohol over‐consumption or viral hepatitis, in contrast to NAFLD, a disease of exclusion. There is a heterogeneity of MAFLD, and the clinical course is influenced by age, diet, alcohol intake, genetics/epigenetics and metabolic health status. The global prevalence of MAFLD is not entirely known but seems to be higher than the prevalence of NAFLD.^13^

In this issue of *United European Gastroenterology Journal*, Váncsa et al take on the important task to investigate a possible association between MAFLD and AP severity.^14^ Given the newly defined criteria of MAFLD, knowledge about a possible correlation between the diseases is of great value for both patient groups. A post hoc cross‐sectional analysis based on over 2000 patients from the AP Registry of the Hungarian Pancreatic Study group was performed. Despite its retrospective design, several important findings are presented. More than one third of all AP patients also suffer from MAFLD and in this group, unsurprisingly, increased rates of comorbidities and alcohol abuse as well as higher levels of body mass index, triglycerides and cholesterol are noticed, despite a significantly lower age. The adjusted multivariate regression model of the whole cohort demonstrates an association between MAFLD and moderately severe AP, although the same connection could not be found for severe AP. However, in further multiple analyses of subgroups investigating individual components of both the metabolic syndrome and the MAFLD criteria, significantly higher odds ratios were found for both severe forms of the disease. Also, the data indicate that metabolic abnormalities and increased odds for severe disease are correlated in a dose‐dependent manner. The findings most likely reflect the complexity of both AP and MAFLD and enhance the importance of the metabolic risk factors.

There is still a lack of sufficient prognostic biomarkers and scoring systems for AP. Continuous work on identifying severity risk factors is thus crucial for optimal management of these patients. Furthermore, unknown intercorrelations between such variables might influence the outcome of the disease. It is also important to bear in mind that comorbidities affecting the severity of AP remain after discharge. As one AP episode increases the risk of recurrent disease, such comorbidities need both observation and treatment. The influence of other diseases on the risk of readmissions and recurrent AP is not well studied, although it is generally accepted that specific etiologies and their treatment affect the risk of recurrent disease and readmissions.^15^ The study by Vancsa et al. demonstrates with clarity that comorbidities and specifically MAFLD have to be taken into account when managing AP. Future studies are called for as the results need to be both validated and assessed. Also, it would be of interest to see if MAFLD increases the risk of recurrent AP and readmissions. Regardless, the data presented here is a large or clear step in the right direction for these groups of patients.

## CONFLICT OF INTEREST STATEMENT

The authors have no conflicts of interest to declare.

## Data Availability

Data sharing not applicable—no new data generated.
